# Effects of Forearm Resistance Exercises on Breast Cancer-Related Lymphedema Using Segmental Bioelectrical Impedance Analysis: A Pilot Randomized Controlled Trial

**DOI:** 10.3390/jcm13237200

**Published:** 2024-11-27

**Authors:** Woo Chul Son, Sang Ah Kim, Ah Hyun Kim, Hawyeong Cheon, Jae Yong Jeon

**Affiliations:** 1 Department of Rehabilitation Medicine, Asan Medical Center, University of Ulsan College of Medicine, Seoul 05505, Republic of Korea; pibiapple@gmail.com (W.C.S.); csa1303@hanmail.net (S.A.K.); apideso@naver.com (A.H.K.); 2 Biomedical Engineering Research Center, Asan Institute for Life Sciences, Asan Medical Center, Seoul 05505, Republic of Korea; cheonhwayeong@gmail.com

**Keywords:** breast cancer, lymphedema, resistance exercise, BIA

## Abstract

**Background**: Breast cancer-related lymphedema (BCRL) reduces the quality of life of patients and limits their activities of daily living. Even though resistance exercises seemed to be safe in BCRL patients, it was still controversial that resistance exercises improve lymphedema. Therefore, we sought to evaluate the effects of forearm-targeted resistance exercises on BCRL using segmental bioelectrical impedance analysis (BIA). **Methods**: This study was a pilot-controlled randomized trial, with patients divided into the intervention and the control group. Both groups received 30 min of complete decongestive therapy (CDT) for 2 weeks. In addition, the intervention group received forearm strengthening training including warm-up and cool-down for an extra 20 min, and the control group received stretching exercises. 5 kHz impedance ratios were assessed by segmental BIA before and after treatments. **Results**: Among the eighteen patients enrolled, ten were assigned to the intervention group, and eight were in the control group. Only the 5 kHz impedance ratio in the forearm segment of the intervention group showed a statistically significant difference. The effect sizes of the groups were 0.71 for the intervention group and 0.93 in the between-group comparison. **Conclusions**: Forearm resistance exercises in patients with BCRL showed a significant decrease in extracellular fluid in the proximal forearm segment when using segmental BIA. Therefore, we suggest that resistance exercises targeting the forearm might be effective in treating lymphedema.

## 1. Introduction

Breast cancer-related lymphedema (BCRL), a common complication of breast cancer surgery, reduces the quality of life of patients and limits their activities of daily living [1]. In the past, it was known, as a principle, not to use the affected arm; however, a series of studies proved that remedial exercises, such as stretching exercises included in complete decongestive therapy (CDT), were effective. Based on these studies, remedial exercises along with CDT were widely performed [2]. Moreover, in recent studies, strengthening exercises, in addition to remedial exercises, improved the patient’s quality of life without increasing the risk of BCRL [3,4,5,6,7]. Some studies even suggest that resistance exercises and physical activity lower the risk of lymphedema [8,9]. Although resistance exercises appeared to be safe, it was still debatable whether they improved lymphedema in patients with BCRL.

Accordingly, various attempts have been made to show that resistance exercises combined with CDT are more useful than CDT alone with conventional manual lymphatic drainage (MLD) massage in patients with lymphedema. Some studies suggest that resistance exercise could reduce edema and have positive effects on the pathophysiology of BCRL [10]. However, most attempts have failed to demonstrate the superiority of resistance exercises over conventional rehabilitation treatment, owing to several reasons [5,11,12]. Furthermore, even in cases where positive effects have been observed, it has been noted that further research is needed on the methods, measurement techniques, and protocols of resistance exercises [10].

One of the reasons for the failure to show the superiority of strengthening exercises is the use of a similar method in previous studies. In many studies, resistance exercises targeting large muscles of the body that were usually used for conditioning exercises in patients with lymphedema were performed and were not associated with lymphedema sites in patients with breast cancer. Moreover, the parameters and evaluation tools used for outcome measures were also similar to those used in CDT studies [1,3,4,5,6,7,11]. Therefore, there was a need for exercises and evaluation tools for precise investigations of the resistance exercises.

Lymphedema was known to occur in the elbow and peripheral areas in patients with BCRL [12,13,14]. If the exercises target the forearm and the muscles around the elbow and not just the large muscles of the entire body, it is expected that changes not confirmed by existing measurement methods, such as arm volume measurement, or simple bioelectric spectroscopy, will be detected. Skeletal muscle pumping in the distal part, particularly through targeted forearm resistance exercises, may propel the lymphatic flow to drain toward the proximal part [15]. By directing lymphatic flow toward the proximal part, lymphedema around the elbow can be improved.

Bioelectrical impedance analysis (BIA), which has been used for several years in recent BCRL research, is a method for measuring the impedance that changes with a difference in composition by flowing current through the body part to estimate the degree of edema. When evaluating BCRL with previously used multifrequency BIA (MFBIA), the entire upper extremity of one side, from the hand to the shoulder, has been evaluated as a single compartment. However, the lymph fluid is not drained equally in all sections of the upper extremity. Therefore, MFBIA may not sufficiently detect this difference compared with sub-limb segmental BIA [12,16,17,18,19]. Accordingly, there have been attempts to measure the impedance by dividing the upper limb into segments, which is known as sub-limb segmental BIA, and it has been shown to be useful in evaluating BCRL compared with MFBIA [20]. Using segmental BIA as the evaluation tool, the effect of the resistance exercises can be detected.

Therefore, in this study, we performed a different mode of resistance exercise targeting the forearm and verified the effects of resistance exercises using segmental BIA in a pilot randomized controlled trial (RCT). Through this, we aimed to examine the effects of applying resistance exercise to small muscle groups using sub-limb segmental BIA, which accounts for the differences in the degree of lymph fluid drainage. First, the single-frequency impedance ratio of segmental BIA was used to determine whether forearm resistance exercises affected the change in water distribution in the targeted area [19,21,22]. Second, we applied both the MFBIA and segmental BIA and compared their abilities to detect the effect of resistance exercises.

## 2. Materials and Methods

### 2.1. Participants

From July 2020 to November 2020, patients with unilateral BCRL who visited the Department of Rehabilitation Medicine of a single center were included. Prior ethical approval was obtained from the Institutional Review Board of Asan Medical Center, Korea (No. 2020-0447). The eligibility criteria were as follows: patients aged ≥18 years who had swelling in a single arm because of breast cancer with International Society of Lymphedema classification stage II; those who underwent breast cancer surgery and had at least two lymph nodes removed; those without evidence of cancer progression; and those without any other disqualification to limit exercise and treatment plans for at least 1 month after exercise. We defined a clinical diagnosis of lymphedema as a difference of 2 cm or >5% in the measured arm circumference [23,24]. We excluded patients who had bilateral breast cancer or trauma; those who had undergone surgery on upper extremities or the neck; and those who could not perform the proposed exercises at least 3 times a week. After the baseline measurement was performed, the patients were randomized into intervention and control groups. Patients were assigned to the two groups through computerized random allocation, which was conducted in an unpredictable manner and concealed from the research staff responsible for determining eligibility.

### 2.2. Intervention

This study was a pilot randomized controlled trial. The intervention group performed a 20 min forearm resistance exercise in each session in addition to CDT for 2 weeks. Each session consisted of 5 min of warm-up and stretching; 10 min of resistance exercises, which mainly targeted the forearm, including wrist flexor exercises using elastic bands (Theraband, Akron, OH, USA) and forearm strengthening exercises using a gripper (Digi-Flex Hand Exerciser, Cando, White Plains, NY, USA), performed in sequence; and 5 min of cooldown. After fixing the elastic bands by stepping on them in the sitting position, the patients performed wrist flexion exercises with the forearm placed on their thighs. After marking the length at which sufficient tension was applied, repetitive exercises were performed at an intensity of 60% of one-repetition maximum (1 RM) during the first week and 80% of 1 RM during the second week, performed as many times as possible. Handgrip exercises were performed in the sitting position with full elbow extension, gradually increasing the intensity. The target score on the Borg scale was 12–16, representing moderate to high intensity. Each exercise was performed in 3 sets of 15 repetitions, and the patients alternately performed the exercises using both arms without a rest period. For the control group, experienced physical therapists performed CDT for 30 min with an additional 20 min of stretching exercises for 2 weeks, 5 times a week. Stretching exercises, combined with deep breathing, were composed of neck rotation, bending, shoulder shrugs, arm movements, wrist bending, and hand stretching. After the first education session, the patients of both groups performed all the exercise sessions under the supervision of the professional physical therapist. The exercises were performed while wearing a bandage or compression garment for both groups, if needed.

### 2.3. Outcome Measure

At the time of enrollment, anthropometric data (e.g., weight, body mass index [BMI], fat mass, and lean mass) and clinical characteristics (e.g., age and sex), and breast cancer-related features (e.g., cancer stage, number of lymph nodes removed, whether chemotherapy or radiotherapy was performed, and chemotherapy agents) were collected.

BIA-related parameters were measured two times using BWA 2.0 (InBody Co., Ltd., Seoul, Republic of Korea) for MFBIA and Inbody M20 (InBody Co., Ltd., Seoul, Republic of Korea) for segmental BIA immediately before and after the treatment sessions. Handgrip, circumference, the EuroQol Visual Analog Scale (EQ-VAS), and EQ-5D-5L were also measured at the time of enrollment and after the last intervention. Handgrip strength was measured using a digital handheld dynamometer after BIA. After measuring twice, the average of the results was used.

### 2.4. Lymphedema Measurement

The circumference of the affected arm was measured in 4 cm intervals from just the distal part of the ulnar styloid process to the axillary fold, and volume was calculated by using Equation (1):

Volume of limbs = Σ (R^2^ + Rr + r^2^) πh/3 (1)

where R is the larger diameter of the limb (the limb circumference × 1/2π), r is the smaller diameter of the limb (the limb circumference × 1/2π), and h is the vertical distance between R and r. Measurement was performed softly while the patient was lying in the supine position using a measuring tape, without tissue indentation [24].

**Figure 1:**
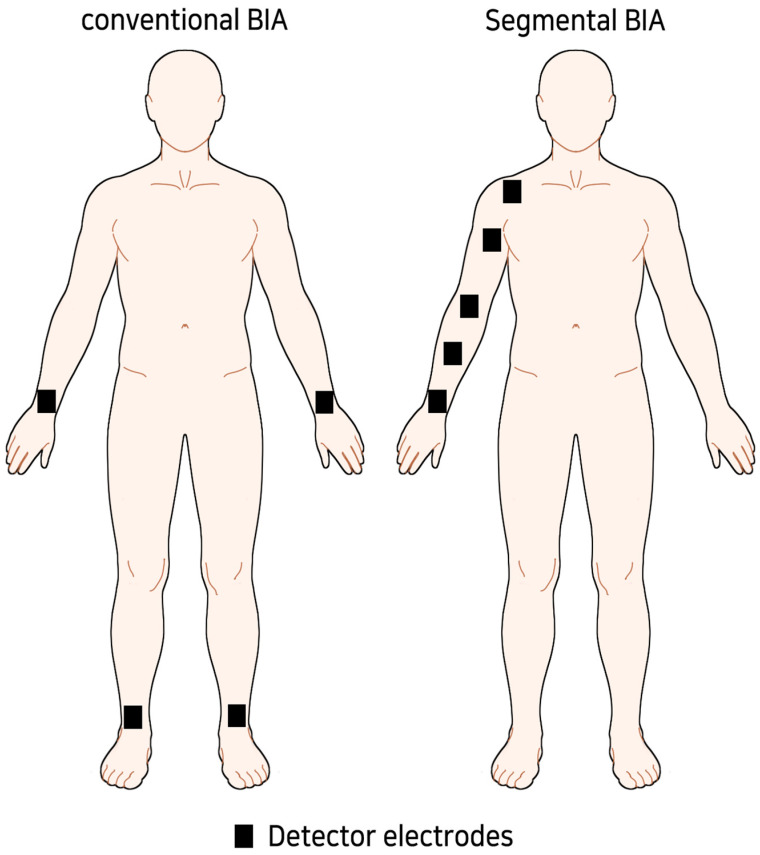
Concept image of sub-limb segmental BIA.

For BIA, the inspector tried to measure in a similar environment as possible. After resting for 5–10 min before measurement, extracellular water (ECW), total body water (TBW), ECW/TBW ratio, and arm body water (ABW) were measured using MFBIA, which were former parameters used for diagnosing BCRL and evaluating the effectiveness of CDT [25,26,27,28]. In the case of segmental BIA, the unilateral arm was divided into four sections based on the ulnar styloid, the midpoint of the ulnar styloid and lateral epicondyle, the lateral epicondyle, the midpoint of the lateral epicondyle and acromioclavicular (AC) joint, and the AC joint (Figure 1). We designated each segment as 1 to 4 from distal to proximal. Each segment was designed to be at least 10 cm in length.

### 2.5. Statistical Analysis

Due to the small sample size, nonparametric tests were used for data analysis. Descriptive statistics for the characteristics of the patients were presented as means (standard deviations) and ranges. The Mann–Whitney test was used to compare the baseline characteristics between the two groups. To confirm the effects of treatment, the Wilcoxon signed-rank test was used for comparison within each group, and the Mann–Whitney test was used for comparison between groups. Effect sizes for the 5 kHz impedance ratio of the segmental BIA were calculated using Cohen’s d.

## 3. Results

### 3.1. Baseline Characteristics of the Patients

**Figure 2:**
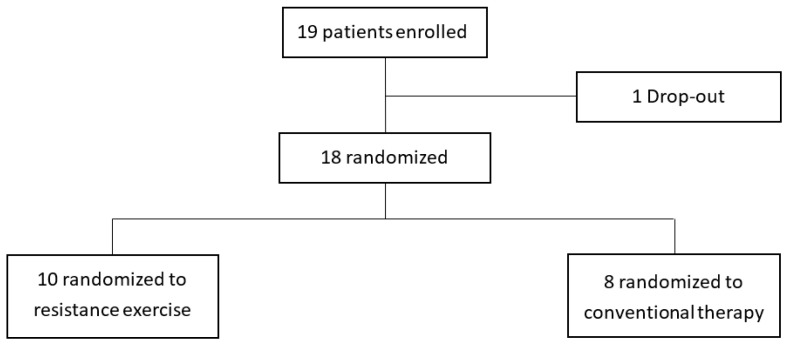
Enrollment of the study population.

Among the patients who visited the outpatient clinic from July 2020 to November 2020, 19 patients who met the inclusion criteria were enrolled. One of them did not come to the hospital for treatment and was dropped out. Ten were assigned to the intervention group, and eight were assigned to the control group (Figure 2). In Table 1, the weight of the control group was 64.8 ± 6.3 kg, which was slightly higher than that of the intervention group (52.6 ± 4.7 kg). Of the 10 sessions, the intervention group attended an average of 8.2 ± 1.1 times, and the control group attended 7.9 ± 0.6 times, showing no significant difference. No adverse events were reported during the exercise sessions in both groups.

### 3.2. Parameters of MFBIA

In Table 2, the ECW/TBW ratio, ECW, and ABW of the affected arm were evaluated within each group. They tended to decrease after the intervention, except for the ECW/TBW ratio, in the control group. There was no significant difference between the two groups in terms of MFBIA parameters.

### 3.3. Parameters of Segmental BIA

**Figure 3:**
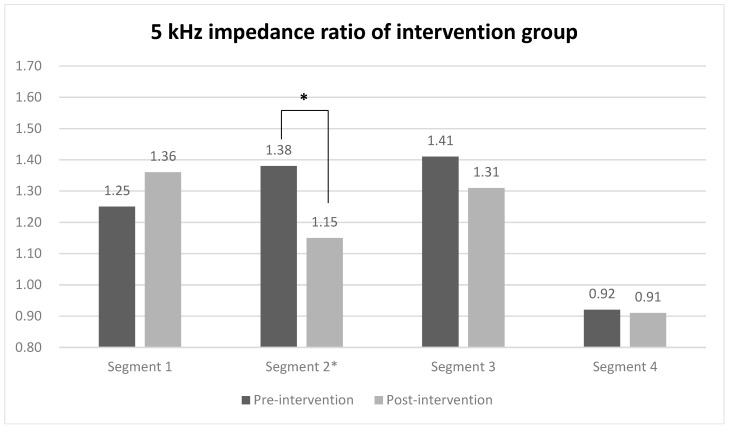

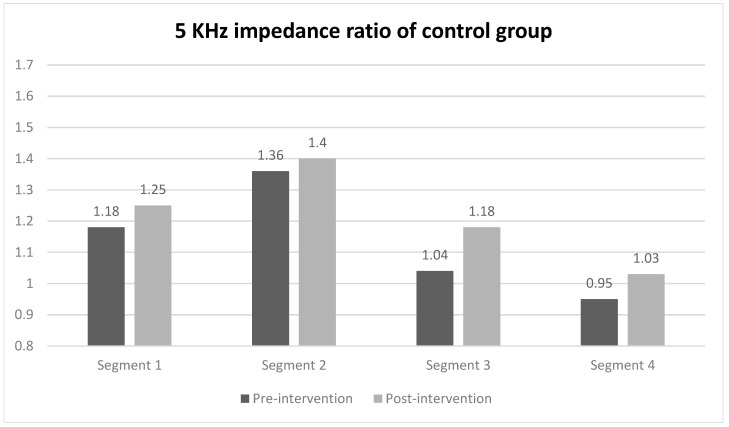
5 kHz impedance ratio of each segment in intervention and control group. Values are presented as median or as indicated. * Wilcoxon signed-rank test.

When evaluating the 5 kHz impedance ratio of each segment using segmental BIA for comparing between groups, a statistically significant difference was observed only in segment 2 (midpoint of the ulnar styloid and the lateral epicondyle to the lateral epicondyle) of the intervention group, with values of 1.38 before the intervention and 1.15 after the intervention (Figure 3). In contrast, the impedance ratios of all segments in the control group did not show statistically significant changes before and after the intervention. The effect sizes of the groups were 0.71 for the intervention group and 0.93 in the between-group comparison (Table 3).

### 3.4. Other Outcomes

In both groups, the volume of the affected limb significantly changed after the sessions. Additionally, the evaluated handgrip strength, EQ-VAS, and EQ-5D-5L showed no significant difference in both groups (Table 4).

## 4. Discussion

This study was a pilot RCT that evaluated the effects of forearm resistance exercises in patients with BCRL using segmental BIA. Previously, some studies had attempted to verify the effects of additionally performing resistance exercises. However, they failed to show the superiority of resistance exercises over conventional MLD or other treatment options, only proving their safety [4,8,11]. Recent studies have reported that resistance exercise shows significant effects on reducing pain or edema in patients with BCRL, but they also recommend further studies using various resistance exercise modes and evaluation tools [10,29]. Considering the lymph drainage system, it seemed that previous evaluation tools were inadequate to catch the efficacy of resistance exercises, particularly those focused on a specific segment. In this study, we attempted to prove it from two perspectives. First, we performed forearm-targeted resistance exercises based on the locations in which lymphedema frequently occurred. Second, we applied segmental BIA. Through this, we could compensate for the difficulty in detecting the composition change or specifically confirming the changes in focal edema [20]. The results of this pilot RCT with the medium intergroup effect size and large within-group effect size of the 5 kHz impedance ratio in segment 2 indicated that it would be useful to imitate this study with a larger sample size.

As mentioned above, lymphedema is known to occur in the forearm and surrounding areas [12,13,14]. Furthermore, there is usually little fat tissue with a relatively large muscle portion, which is advantageous for determining the effectiveness of resistance exercises. Hence, the forearm may be one of the most suitable body parts for proving the effectiveness of resistance exercises. It is also possible that muscle contraction through forearm resistance exercises improved subfascial lymph drainage and demonstrated more effective lymph drainage [13,30]. In other words, resistance exercises that generate counterpressure between the muscle and a compression garment or skin could create similar effects to MLD. Therefore, resistance exercises targeting the forearm with CDT could be effective in improving lymphedema, particularly in patients with BCRL.

In this study, the 5 kHz impedance ratio using segmental BIA significantly improved in the proximal forearm section. It showed a medium effect size in the intervention group and a large effect size in the between-group comparison. In previous studies validating resistance exercise, some used bioimpedance spectroscopy (BIS) to measure total body water. However, this approach only measures total volume and classifies the entire limb as a single segment, which presents a limitation in distinguishing specific components, such as lymphedema and muscle mass, in the desired area [9]. This may result in the relative growth of muscle mass, masking the effects of the exercise. Therefore, sub-limb segment analysis using specific impedance ratios is presumed to be more useful from certain perspectives. The proximal forearm was the actual targeted area of the resistance exercises performed, and the resistance exercises performed mainly on a specific area were effective in improving interstitial fluid discharge in the relevant area. This finding was consistent with those of previous studies that indicated that lymphedema seemed not to have a uniform distribution and might be mainly occurring around the elbow in relation to the lymphatic drainage system along the forearm [12,13,14]. Considering these findings, segmental BIA could be a useful monitoring tool for patients with BCRL when performing resistance exercises.

ECW and ABW tended to decrease in both groups, but MFBIA parameters yielded no significant results. Given that both groups performed CDT, these results differed from previous studies that found a statistically significant change in MFBIA parameters after CDT in patients with BCRL [1,3]. We assumed that this was because of the small number of enrolled patients and MFBIA’s relatively low sensitivity compared with segmental BIA. If the exercise is performed by targeting the forearm and the study is carried out with a large number of patients, we can expect that MFBIA will also aid in proving the effect of forearm-targeted resistance exercises.

Handgrip strength did not change significantly. Handgrip strength in the intervention group did not improve significantly when compared with the control group. Forearm muscles are usually composed of type 1 muscle fiber, and we assumed that 2 weeks of exercises seemed to be relatively short for functional change, considering that at least 4 weeks of exercises were performed in other studies [3,31]. However, as shown in Table 4, although not statistically significant, there was an overall increasing trend compared to before the intervention. This suggests that in a large population study, the results could be statistically significant. Further studies with a longer exercise schedule are required.

### Study Limitations

This study has several limitations. First, as a pilot study, the number of enrolled patients was small; therefore, further study with a larger study population is required. Second, it was possible that incomplete data were derived because of the instability of the equipment used. Third, because the parameters of MFBIA were not statistically significant in the within-group comparisons, we could not perform the comparisons between the groups. Fourth, since the weight difference between the two groups was statistically significant, the results could be biased, considering that weight could affect lymphedema. Finally, because of the relatively short intervention period, only the time point immediately after 2 weeks of exercises was evaluated; therefore, the long-term outcomes could not be evaluated. Follow-up studies are needed to determine whether the effect of exercises continues and examine the effect of resistance exercises focusing on partial segments on the prognosis of BCRL.

## 5. Conclusions

Forearm resistance exercises in patients with BCRL showed a significant decrease in extracellular fluid in the proximal forearm segment when using segmental BIA. Therefore, resistance exercises targeting the forearm might be effective in treating lymphedema with appropriate use of segmental BIA.

## Figures and Tables

**Table 1:** Baseline characteristics of the patients.

Characteristic	Intervention (*n* = 10)	Control (*n* = 8)	*p*-Value
**Age** (years)	48.9 ± 8.4	53.3 ± 6.0	0.31
**Weight**	52.6 ± 4.7	64.8 ± 6.3	<0.01 *
**Height**	157.0 ± 4.9	159.5 ± 3.2	0.31
**BMI**	21.5 ± 2.3	25.5 ± 2.5	<0.01 *
**Months since operation** (months)	64.4 ± 53.0	86.9 ± 100.7	0.79
**No. of nodes removed**	20.2 ± 8.1	26.5 ± 5.5	0.82
**Chemotherapy**	10/10 (100%)	8/8 (100%)	>0.99
**Radiation**	5/10 (50%)	5/8 (62.5%)	0.74
**Current receipt of drugs**			0.60
Tamoxifen	3/10 (30%)	2/8 (25%)	
Aromatase inhibitors	0/10 (0%)	3/8 (37.5%)	
Xeloda	2/10 (20%)	1/8 (12.5%)	
**Cancer stage**			0.35
1	0/10 (0%)	1/8 (12.5%)	
2	2/10 (20%)	3/8 (37.5%)	
3/4	2/10 (20%)	2/8 (25%)	
Unknown	6/10 (60%)	2/8 (25%)	
**Attendance rate**	8.2 ± 1.1	7.9 ± 0.6	0.28

Values are presented as mean ± SD or as indicated. * Mann–Whitney test. Abbreviations: BMI, body mass index; No., number.

**Table 2:** Parameters from MFBIA.

Characteristic	Intervention (*n* = 10)		*p*-Value	Control (*n* = 8)		*p*-Value
	First	Last		First	Last	
**ECW/TBW**	0.40 (0.38–0.41)	0.39 (0.38–0.40)	0.17	0.39 (0.39–0.41)	0.40 (0.39–0.41)	0.36
**ECW** **(L)**	0.58 (0.48–0.77)	0.55 (0.44–0.69)	0.20	0.65 (0.51–0.83)	0.60 (0.49–0.82)	0.14
**ABW(L)**	1.48 (1.21–1.88)	1.40 (1.16–1.73)	0.29	1.54 (1.30–2.05)	1.53 (1.26–2.01)	0.09

Values are presented as median (minimum–maximum). Abbreviations: ECW, extracellular water; TBW, total body water; ABW, arm body water.

**Table 3:** 5 kHz impedance ratio of each segment by segmental BIA.

Segment		Pre-Intervention Ratio	Post-Intervention Ratio	*p*-Value	Effect Size	
					Within	Inter
**1**	**Intervention**	1.25 (0.88–1.87)	1.36 (0.99–2.16)	0.29		
	**Control**	1.18 (0.89–2.00)	1.25 (0.87–1.79)	0.67		
**2**	**Intervention**	1.38 (1.00–2.38)	1.15 (0.95–2.03)	0.01 *	0.71	0.93
	**Control**	1.36 (0.86–1.97)	1.40 (0.75–1.76)	0.21	0.12	
**3**	**Intervention**	1.41 (0.99–1.98)	1.31 (0.91–1.74)	0.24		
	**Control**	1.04 (0.65–1.45)	1.18 (0.71–1.68)	0.12		
**4**	**Intervention**	0.92 (0.64–1.12)	0.91 (0.70–1.40)	0.88		
	**Control**	0.95 (0.74–1.16)	1.03 (0.75–1.11)	0.33		

Values are presented as median (minimum–maximum) or as indicated. * Wilcoxon signed-rank test.

**Table 4:** Volume of the affected limb, strength, and EQ-VAS, 5D-5L.

Characteristic	Intervention (*n* = 10)		*p*-Value	Control (*n* = 8)		*p*-Value
	First	Last		First	Last	
**Volume of** **the affected limb**	1616.4 (1375.5–1863.5)	1594.9 (1375.5–1863.5)	<0.01 *	2116.7 (1520.8–2645.7)	1992.1 (1520.8–2508.2)	0.01 *
**Grip strength**	21.0 (13.6–24.6)	21.7 (14.1–25.1)	0.07	18.3 (14.7–21.1)	19.0 (15.3–23.5)	0.14
**EQ-VAS**	77.5 (50–95)	80.0 (60–100)	0.22	70.0 (20–90)	72.5 (70–100)	0.07
**EQ-5D-5L**	7.5 (6–10)	8.0 (5–9)	0.71	6.5 (5–18)	7.0 (5–13)	0.14

Values are presented as median (minimum–maximum) or as indicated. * Wilcoxon signed-rank test.

## Data Availability

The data that support the findings of this study are available from the corresponding author upon reasonable request.
